# New insights into long noncoding RNAs and their roles in glioma

**DOI:** 10.1186/s12943-018-0812-2

**Published:** 2018-02-19

**Authors:** Zixuan Peng, Changhong Liu, Minghua Wu

**Affiliations:** 1 Hunan Provincial Tumor Hospital and the Affiliated Tumor Hospital of Xiangya Medical School, Central South University, Changsha, Hunan 410006 China; 2 Cancer Research Institute, School of Basic Medical Science, Central South University, Key Laboratory of Carcinogenesis and Cancer Invasion, Ministry of Education, Key Laboratory of Carcinogenesis, Ministry of Health, Changsha, Hunan 410078 China

**Keywords:** LncRNAs, Non-coding RNA, Glioma, Biomarker, Therapeutic targets

## Abstract

Glioma is one of the most prevalent types of primary intracranial carcinoma with varying malignancy grades I–IV and histological subtypes, including astrocytomas, glioblastoma multiform (GBM), oligodendrogliomas and mixed tumors. Glioma is characterized by rapid cell proliferation and angiogenesis, and the WHO grade IV glioblastoma, which is highly malignant with poor prognosis because GBM stem-like cells (GSCs) are resistant to conventional therapy and easily recrudescent, accounts for the majority of gliomas. Consequently, investigations exploring the accurate molecular mechanisms and reliable therapeutic targets for gliomas have drawn extensive attention.

Based on the increasing amount of functional lncRNAs aberrantly expressed in glioma tissues and cell lines, lncRNAs might be critical for glioma initiation, progression and other malignant phenotypes. This review summarizes the latest insights into the lncRNA field and their functional roles in glioma, therefore evaluating the potential clinical applications of lncRNAs as prospective novel biomarkers and therapeutic targets.

## Background

LncRNAs comprise a wide variety of RNA transcripts of sizes greater than 200 nucleotides (nt) that lack significant protein-coding capacity [1], typically with 5’m7G caps and 3′ poly(A) in tails, similar to the structure of mRNAs [2], but are more tissue-specific and dynamic than mRNAs, suggesting that these molecules have distinct biological roles [3]. LncRNAs were initially implicated in the epigenetic regulation of X chromosome inactivation during embryogenesis [4]. The last several years has seen a steady increasing interest in identifying lncRNAs and understanding their regulatory functions in almost all aspects from gene expression to protein translation and stability [5], either *in cis* (regulate neighboring genes) or *in trans* (regulate distant genes) [6]. (Table 1).

**Table 1:** Examples of lncRNA functional mechanisms

Primary mechanism	LncRNA	Detailed mechanism	Function	Reference
Chromatin modification	LncPRESS1	Disrupt deacetylation of H3K56 by sequestering SIRT6 from chromatin	Safeguard the stem cells pluripotency	[17]
	NEAT1	Bind to EZH2 and mediate the trimethylation of H3K27 in their promoters.	Promote glioma cell growth and invasion by increasing β-catenin nuclear transport	[25]
	HOTTIP	Maintain gene transcription by binding to adaptor WDR5	Promote hepatic carcinoma tumorigenesis and disease progression	[26]
	FOXC1	Stabilize enhancer–promoter looping	Involved in many regulated programs in breast cancer	[15]
	NBAT-1	Activate the neuronal-specific transcription factor NRSF/REST	Contribute to aggressive neuroblastoma	[51]
Alternative splicing	SPA1, SPA2	Bind to target mRNAs and sequester multiple RBPs to regulate alternative splicing	Involved in Prader-Willi syndrome pathogenesis	[21]
	LncRNA BC200	Bind to Bcl-x pre-mRNA and recruit splicing factor hnRNP A2/B1	Modulate Bcl-x alternative splicing in breast cancer	[28]
mRNA stability/modification	KRT7-AS	Form “lncRNA-mRNA” protective duplex at overlapping region	Promote gastric cancer cell migration	[27]
	MEG3	Act as RNA scaffold to form PTBP1-mediated Shp RNA decay	Promote hepatocirrhosis in hepatocellular carcinoma	[29]
	FOXM1-AS	Facilitate interaction of ALKBH5 and FOXM1 mRNA to demethylate FOXM1 mRNA	Enhance self-renewal and tumorigenesis of glioblastoma stem-like cells	[50]
miRNA sponge	TP73-AS1	Increase HMGB1 expression by sponging miR-142	Promote glioma proliferation and invasion	[76]
	CASC2	Inhibit miR-181a activity with RISC complex participation	Sensitize TMZ-resistant glioma cells to TMZ	[45]
Change protein activity/localization	HULC	Scaffold of kinase and YB-1 to promote YB-1 phosphorylation	Accelerate the translation of tumorigenesis mRNAs in hepatocellular carcinoma	[31]
	LINK-A	Facilitate BRK-dependent HIF1α phosphorylation	Promote breast cancer glycolysis reprogramming and tumorigenesis	[18]
	SNHG5	Trap MTA2 in the cytosol and prevent it translocation into nucleus.	Suppress gastric cancer progression	[32]
Encode functional micropeptides	LINC00948	Translate myoregulin, which inhibit sarcoplasmic reticulum Ca2 + -ATPase	Regulate Ca2+ uptake and skeletal muscle contractility	[34]
	LINC00961	Generate SPAR, which interact with lysosomal v-ATPase	Modulate skeletal muscle regeneration after injury	[35].
Intercellular communication	LncARSR	Transmit from sunitinib-resistance cell to sensitive cells	Confer Sunitinib resistance to sensitive cells in renal cancer	[39]
	H19	Transmit from cancer stem-cell-like (CSC) to endothelial cells	Promote angiogenesis in hepatic carcinoma	[40]
	ENST00000444164 ENST0000043768	Transmit from epithelial ovarian cancer (EOC) to endothelial cells	Promote epithelial ovarian cancer cells migration	[41]

In accordance with their significant roles in extensive biological processes, lncRNAs have been implicated in the onset and progression of cancer malignancy, including glioma, such as stemness, proliferation, angiogenesis and drug resistance [7, 8]. Moreover, aberrant lncRNA expression profiles in clinical glioma specimens correlates with malignancy grade and histological differentiation, which have important clinical implications in glioma diagnosis of sub-classification [9] and prognostication [10, 11]. In addition, lncRNAs transmitted from cell to cell participate in intercellular communications for maintaining microenvironment homeostasis or mediating tumor metastasis. This review summarizes the most up-to-date knowledge regarding how lncRNAs function at the molecular level and their implications in the areas of glioma research and therapy.

## LncRNA categories and structures

### LncRNA classification based on genomic location

**Fig. 1:**
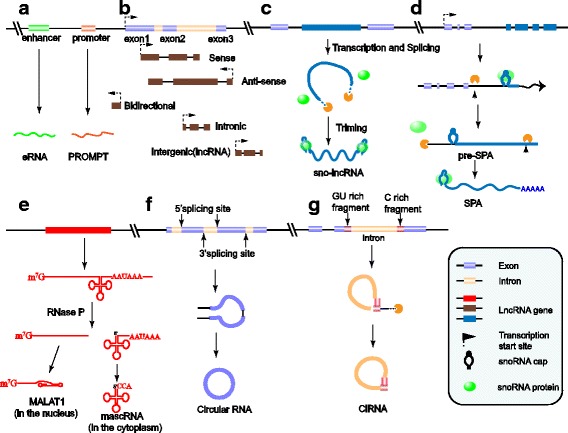
Schematic representation of lncRNA species and structures. **a** LncRNAs transcribed from functional DNA element regions: promoter upstream transcript (PROMPTs), enhancer-associated RNA(eRNAs); (**b**) Mainstream five categories based on genomic location and transcript orientation of lncRNA: (1) sense lncRNAs and (2) antisense lncRNAs, overlapping and transcribed from the same or opposite strand of protein-coding genes, (3) bidirectional lncRNAs, typically transcribed in the opposite direction of its neighboring protein-coding gene (less than 1 kb away) [13], (4) intronic lncRNAs, transcribed entirely from introns [14], (5) intergenic lncRNAs (lincRNAs), transcribed from genomic interval [1]; (**c**, **d**) LncRNAs characterized by snoRNA caps: Sno-lncRNAs have snoRNA caps at both ends, while SPA-lncRNAs possess 5’ snoRNA only; (**e**) LncRNAs characterized by tRNA-like extremities, with the 3′ end alternatively processed by ribonuclease P (RNase P), e.g., MALAT1 and NEAT1_2; (**f**, **g**) LncRNAs with close circular structures: derived from back-splicing of exons named circular RNAs, and ciRNAs are produced from intron lariats.

LncRNAs can be classified into the following five categories according to their genomic location relative to neighboring protein-coding genes [12]: sense, antisense, bidirectional [13], intronic [14] and intergenic (lincRNAs) [1] (more detail in Fig. 1). Moreover, the promoter upstream transcripts (PROMPTs) and enhancer-associated RNAs (eRNAs), transcribed from promoters or enhancers, show some similarities in functional mechanism with regulatory DNA elements [12]. For instance, eRNAs likely facilitate enhancer and promoter interactions and thereby activate target genes [15].

### LncRNA categories based on subcellular localization

Since the functional mechanism of lncRNAs depends on their subcellular localization [16], lncRNAs can also be grouped into nuclear or cytoplasmic categories [5]. LncRNAs that exhibit distinct nuclear localization patterns typically alter gene transcription via transcriptional interference [17] and chromatin remodeling [15], whereas lncRNAs exported to the cytoplasm perform regulatory roles by mediating RNA processing, affecting mRNA stability or directly regulating protein function [18].

### New LncRNA species based on unique structures

Given that lncRNAs structures are associated with their stability and functional mechanisms [16], the identification of lncRNA secondary structures will contribute to their applications in clinical medicine [19]. Most linear lncRNAs have 5’m7G caps and 3′ poly(A) tails, similar to mRNAs, while some noncanonical terminal lncRNAs processed with unique transcription patterns can be defined as new RNA species [20]. Sno-lncRNAs and SPA-lncRNAs [21] are characterized by snoRNA caps at both ends or only at the 5′ terminus, having longer half-lives than other lncRNAs. In addition, the RNase P-mediated processing of lncRNAs results in tRNA-like structures at the extremity and increased stability in the cytoplasm, for which a typical example is MALAT1 [22]. As for lncRNAs that exist in loop form, there are circular RNAs [23] and circular intronic RNAs (ciRNAs) [24] produced from the back-splicing of exons or introns respectively (Fig. 1).

## Functional mechanisms of lncRNAs in glioma and other cancers

### LncRNAs regulate genome activity

Unsurprisingly, many lncRNAs participate in the epigenetic regulation of gene expression by recruiting chromatin modifiers (e.g., EZH2/PRC2 [25] and WDR5/TrxG [26]) to a specific genomic location as a ***Scaffold***, segregating chromatin modifiers away from their regulatory targets as a ***Decoy*** [17] or mediating the 3D organization of chromosomes [15].

In glioma, NEAT1 mediates the trimethylation of H3K27 in the promoter region of WNT/β-catenin pathway negatively regulated factors (Axin2, ICAT and GSK3B) by physically interacting with the PRC2 subunit EZH2 as a ***Scaffold***, thereby resulting in WNT/β-catenin pathway activation [25]. During hepatocarcinogenesis, lncRNA HOTTIP directly interacts with the trithorax group (TrxG) protein WDR5 and induces an open DNA-chromatin configuration to target WDR5/MLL complexes, driving H3K4 trimethylation and thus promoting the transcription of HOXA locus genes [26]. Increased by estradiol (E2) stimulation in breast cancer, FOXC1 contributes to E2-dependent gene activation by promoting the 3D contacts between the specific promoter and enhancer cluster and increasing the strength of enhancer–promoter looping [15].

### LncRNAs related to posttranscriptional regulation

Aside from regulating the alternative splicing [21] and stability [27] of mRNAs, the competing endogenous RNAs (ceRNAs) or miRNA sponge mechanism are also implicated in lncRNAs post-transcriptional regulation. (Detailed in Table 2).

**Table 2:** Deregulated lncRNAs in glioma

	LncRNA	Mechanisms	Function	References
Up-regulated	NEAT1	Bind to EZH2 and mediate H3K27me3 in target promoters.	Promote glioma cell growth and invasion	[25]
	HOTAIR	Interact with the PRC2 complex	Promotes glioblastoma cell cycle progression	[52]
		HOTAIR/miR-326/FGF1 axis	Promotes malignant biological behaviors of glioma cells	[43]
		Bind to miR-148b-3p as ceRNA and enhance tight junction	Decrease the permeability of BTB in glioma	[62]
	TUG1	Inhibit miR-144 and reverse miR-144 effect on occludin, ZO-1 and claudin-5	Regulate BTB permeability in glioma	[63]
	FOXM1-AS	Facilitate interaction of ALKBH5 and FOXM1 mRNA to demethylate FOXM1 mRNA	Enhance self-renewal and tumorigenesis of glioblastoma stem-like cells	[50]
	CRNDE	Modulate the mTOR signaling pathway.	Promote glioma cell growth and invasion	[42]
		Attenuate miR-384/PIWIL4/STAT3 axis	Facilitate glioma cells proliferation and invasion, while inhibited cells apoptosis	[30]
		Bind to miR-136-5p as ceRNA, thereby protecting Bcl-2 and Wnt2	Enhance migratory and invasive capacities of glioma cells	[77]
	H19	Up-regulate the VASH2 expression by decreasing miR-29a.	Promote glioblastoma cell invasion, angiogenesis and tube formation	[57]
		Derive miR-675, which directly suppresses CDK6	Promote glioma cell proliferation and migration	[78]
	SOX2OT	Bind to both miR-194-5p and miR-122, reverse SOX3 expression	Promote proliferation, migration and invasion of GSCs	[79]
	ECONEXIN	Increase TOP2A by sponging miR-411-5p	Maintain aggressive proliferation of glioma cells	[46]
	HCP5	Form HCP5-miR-139-RUNX1 positive feedback loop	Induce proliferation, migration and invasion of glioma cells	[53]
	XIST	Increase the expression of ZO-2 and transcription factor FOXC1 as miR-137 sponge	Decrease blood–tumor barrier permeability and promote glioma angiogenesis	[58]
		Form RNA induced silencing complex RISC with miR-152	Promote GSC proliferation, migration and invasion	[48]
		Downregulate miR-429 as sponge	promote glioma tumorigenicity and angiogenesis	[59]
	CASC2c	Compete to combine miR-101 by repelling CPEB1	Promote the malignant characteristic of astrocytoma cells	[80]
Down-regulated	NBAT-1	Suppress the neuronal-specific transcription factor NRSF/REST	Impair proliferation and increase differentiation of neuroblastoma	[51]
	TUG1	Recruit polycomb to methylate locus-specific histone H3K27	Maintain stemness features of GSCs	[47]
		Induce the activation of caspase-3 and caspase -9	Induced glioma apoptosis	[81]
	GAS5	Form GAS5/miR-196a-5p/FOXO1 positive feedback loop	Suppress glioma stem cells proliferation, migration, and invasion	[44]
		Increase the expression of bmf and Plexin C1 by downregulating miR-222	Suppress glioma malignancy and tumor size	[82]
	MALAT1	Downregulate miR-155 expression	Suppress the invasion and proliferation of glioma cells	[54]
		Attenuate ERK/MAPK-mediated growth and MMP2-mediated invasiveness.	Suppress the growth and invasion of glioma cells	[56]
		Up-regulate EMT related proteins	Decrease the sensitivity of glioblastoma cells to TMZ	[65]
	CASC2	Interact with miR-181a, increase the expression of PTEN	Inhibit glioma cells proliferation and amplify TMZ sensibility	[45]

During mRNA alternative splicing, lncRNA BC200 recruits splicing factors hnRNP A2/B1 to Bcl-x pre-mRNA and contributes to breast cancer pathogenesis [28]. From the perspective of regulating mRNA stability, lncRNAs may either form protective “lncRNA-mRNA” duplex to stabilize mRNAs [27] or, conversely, facilitate mRNA degradation by recruiting RNA-binding proteins, such as PTBP1 to target pre-mRNA [29]. Moreover, LncRNAs can regulate the efficiency of mRNA translation. Upon energy stress, FILNC1 interacts with AUF1, a c-Myc mRNA-binding protein, and sequesters AUF1 from binding c-Myc mRNA, leading to the downregulation of c-Myc protein. Cytoplasmic lncRNAs can participate in the ceRNA regulatory network and act as endogenous miRNA sponges through direct base pairing. In high-grade GBM, the up-regulation of CRNDE competitively binds to miR-384 and decreases the availability of miR-384 to its downstream target genes (STAT3, cyclin D1 and Bcl-2), thus abrogating the suppression of miR-384 downstream genes [30].

### LncRNAs in protein modification and anchoring

LncRNAs can also directly bind proteins essential for a signaling pathway and modulate their functions. A novel mechanism by which lncRNA plays an oncogenic role through modulating the phosphorylation status of its interaction protein is demonstrated in many cancers. LINK-A recruits breast tumor kinase (BRK) to phosphorylate Tyr565 of hypoxia-inducible factorα (HIF1α), and the activated HIF1α signaling promotes breast cancer glycolysis reprogramming [18]. *HULC* specifically binds to YB-1 (translationally inactive ribonucleoprotein particle) and promotes YB-1 phosphorylation, which leads to the release of YB-1 from its bound mRNA. Consequently, the translation of silenced oncogenic mRNAs (cyclin D1, cyclin E1, and MMP3) in hepatocellular carcinoma would be activated [31].

Moreover, lncRNA SNHG5 can trap MTA2 in the cytoplasm, preventing MTA2 translocation into the nucleus and interfering with nucleosome remodeling. The significantly downregulated SNHG5 in gastric cancer, thereby promoting gastric cancer progression [32].

### LncRNAs encode functional micropeptides

Recently, some lncRNAs have been shown to encode functional micropeptides based on small-ORFs [33]. For instance, micropeptides translated from lncRNAs regulate skeletal muscle regeneration or contractility by interacting with sarcoplasmic reticulum Ca^2+^-ATPase (SERCA) [34], lysosomal v-ATPase [35], or displacing SERCA inhibitors [36]. Moreover, the micropeptides encoded by lncRNA HOXB-AS3 competitively bind to hnRNPs, suppressing glucose metabolism reprogramming in colon cancer [37].

### Exosome-transmitted lncRNAs function as intercellular communicator

LncRNAs play roles in intercellular communication, wherein the release of lncRNAs into the recipient cells can modulate cell phenotypes through the regulation of target signaling pathways [38]. Studies have shown that lncRNAs secreted from drug-resistant renal carcinoma cells via exosomes can transform adjacent drug-sensitive cells into a resistant phenotype [39]. Moreover, exosome-transmitted lncRNAs can regulate the tumor microenvironment and mediate the communication of heterogeneous cells [40, 41]. Through the release of exosomes containing lncRNA H19, CD90+ liver cancer cells can modulate the phenotypes of endothelial cells, promoting angiogenesis and cell-to-cell adhesion [40].

## LncRNAs regulate glioma malignant phenotypes

**Fig. 2:**
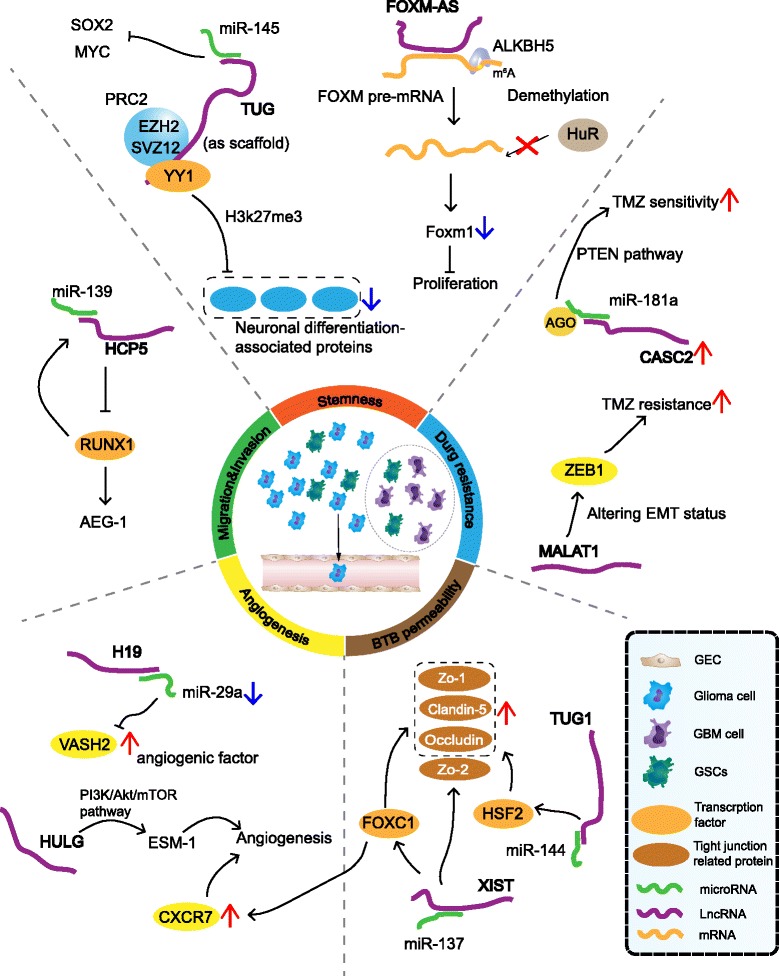
Functional roles of lncRNAs in glioma malignancy and chemoresistance. LncRNAs contribute to five major malignant phenotypes of glioma by targeting different cell species: general glioma cells, glioma-associated endothelial cells, GBM cells, and GBM stem-like cells. Selected examples of lncRNAs and their molecular partners or genomic targets are shown for stemness, drug resistance, BTB permeability, angiogenesis and motility cancer phenotypes.

Accumulating evidence has demonstrated that many dysregulated lncRNAs in glioma are closely related to tumorigenesis, metastasis and prognosis or therapies [7, 8] (Fig. 2 and Table 2). LncRNAs can function as molecular signaling mediators, modulating the expression of a certain set of genes and corresponding signaling pathways (e.g., the NEAT1-WNT/β catenin pathway [25] and CRNDE- mTOR signaling [42]).

The majority of the glioma-associated lncRNAs serve as “miRNA sponges” to quench miRNA activity (e.g., HOTAIR/miR-326 [43], Gas5/miR-196a-5p [44], CASC2/ miR-181a [45] and ECONEXIN /miR-411-5p [46]).

### LncRNAs related to glioma stemness

Recent studies have shown that lncRNA TUG1 maintains the stemness of GSCs through epigenetically suppressing multiple neuronal differentiation-associated genes. TUG1 physically interacts with PRC2 components (EZH2 and SUZ12) and transcription factor YY1 as a protein scaffold, promoting the locus-specific methylation of histone H3K27 [47]. LncRNA GAS5 suppresses GSC proliferation, migration and invasion by binding to onco-miR-196a-5p and upregulating the downstream FOXO1. Interestingly, the increased FOXO1 promotes GAS5 transcription, thus forming a positive feedback loop [44]. LncRNA XIST promotes the proliferation and inhibits the apoptosis of GSCs. More than directly binding to miR-152 as a sponge, XIST is likely in the same RNA-induced silencing complex (RISC) with miR-152, thus abrogating miR-152 downstream pathways [48].

Post-transcriptional mRNA modification provides an additional regulation of mRNA stability, and N6-methyladenosine (m6A) is the most prevalent internal modification on mRNAs [49]. LncRNA FOXM1-AS promotes the interaction of FOXM1 nascent transcripts with RNAm6A demethylase ALKBH5, leading to the demethylation and elevated expression of FOXM1. The elevated expression of transcription factor FOXM1 then facilitates GSC self-renewal and tumorigenesis [50].

### LncRNAs related to glioma proliferation and migration

Regulated by EGFR pathway activity, NEAT1 is critical for glioma cell growth and invasion through the WNT/β-catenin pathway. NEAT1 mediates the H3K27 trimethylation of three WNT/β-catenin pathway negatively regulated factors, thereby increasing β-catenin nuclear transport and rekindling the WNT/β-catenin pathway [25]. Considering that NBAT-1 is expressed at low levels in high-risk neuroblastoma, NBAT-1 acts as a scaffold for EZH2 recruitment and represses NBAT-1/EZH2 target genes, which are involved in neuroblastoma progression [51]. It was demonstrated that the 5′ domain of HOTAIR can bind to the EZH2 and promote glioblastoma cell cycle progression [52].

The oncogene ECONEXIN is predominantly located in the cytoplasm and interacts with miR-411-5p via two binding sites, regulating TOP2A by sponging miR-411-5p in early glioma tumorigenesis [46]. Furthermore, lncRNA HCP5 sponge miR-139 to up-regulate RUNX1, and then RUNX1 feedback promoted HCP5 expression by binding to HCP5 promoters, creating a HCP5/miR-139/RUNX1 positive feedback loop to regulate the malignant behavior of glioma cells [53]. As a glioma-suppressor, MALAT1 inhibits cell viability by downregulating miR-155 expression [54], and a similar mechanism was also observed in the “TUSC7-miR-23b” axis [55]. The knockdown of CRNDE decreases the protein level of PIWIL4, a target of miR-384, which leads to glioma regression in vivo [30].

MALAT1 play a tumor-suppressive role in glioma by attenuating ERK/MAPK-mediated growth and MMP2-mediated invasiveness. The decreased level of MALAT1 in glioma significantly increased tumorigenicity in both subcutaneous and intracranial human glioma xenograft models [56].

Angiogenesis is a universal characteristic of blood vessel-rich glioma progression that facilitates proliferation and migration. In glioma microvessels, the overexpression of H19 induces glioma-associated endothelial cells (GECs) proliferation, migration and tube formation via increasing the expression of angiogenic factor VASH2 as a miR-29a sponge [57]. Up-regulated XIST can facilitate glioma angiogenesis by increasing the promoter activity of chemokine receptor 7b (CXCR7) [58], or down-regulating miR-429 as a molecular sponge [59]. LncRNA HULC regulates ESM-1 via the PI3K/Akt/mTOR signaling pathway and hence plays a pro-angiogenesis role in human gliomas [60].

### LncRNAs related to invalid chemotherapy

Conventional treatment of glioma includes surgical resection followed by radiotherapy and temozolomide (TMZ) chemotherapy. Invalid drug administration in glioma is mainly caused by either the poor permeability of the blood–tumor barrier (BTB), which invalidates adjuvant chemotherapy [61] or the acquired chemo-resistance of tumor cells.

Modulating the biological behaviors of glioma vascular GECs is an important aspect of lncRNAs influencing therapeutic effect. For example, HOTAIR decreases BTB permeability via binding to miR-148b-3p, which further increasing tight junction-related proteins (ZO-1, occludin and claudin-5) in GECs by regaining upstream stimulating factor 1 (USF1) [62]. Similarly, the aberrant high expression of lncRNA XIST in GECs reduces BTB permeability by increasing tight junction-related proteins (ZO-1, ZO-2 and occludin) [58]. LncRNA TUG1 is also up-regulated in endothelial cells and regulates BTB permeability through targeting miR-144 [63]. Some researches demonstrated that the knockdown of MALAT1 resulted in the increased permeability of BTB, which might contribute to the reworking of chemotherapeutics [61].

TMZ is a first-line chemotherapeutic drug most widely used for treating gliomas, whereas drug resistance is largely responsible for the failure of glioma chemotherapy [64]. Research has shown that cells with epithelial-to-mesenchymal transition (EMT) characteristics tend to be more resistant to anticancer drugs. LncRNA MALAT1 is up-regulated in multidrug-resistant glioblastoma cell lines and decreases the sensitivity of glioblastoma cells to TMZ by up-regulating EMT-related proteins (ZEB1, Snail and SLUG) [65]. LncRNA CASC2 up-regulates the tumor suppressor PTEN through directly inhibiting miR-181a, thus amplify TMZ sensibility of glioma cells [45].

## Promising biomarker: lncRNAs in diagnostic or prognostic applications

The aberrant lncRNA expression profiles in clinical specimens correlates with malignancy grade and histological differentiation, which have important clinical implications in the glioma diagnosis of sub-classification [9] and prognostication [10, 11]. One of the distinctive features of lncRNAs is their highly tissue- and cell type-specific expression patterns [3], which could accurately classify different subtypes of glioma and predict responses to treatments.

One-way analysis of variance (ANOVA) indicates a markedly significant difference in HOXA11-AS expression between the four GBM subtypes in CGGA mRNA microarray datasets, and HOXA11-AS expression in classical and mesenchymal subtypes is higher than that in neural and proneural subtypes, suggesting that HOXA11-AS might serve as a biomarker for identifying glioma molecular subtypes [9]. The up-regulated oncogene CRNDE is correlated with larger tumor size (*p* = 0.011), higher WHO grade (*p* = 0.001), and recurrence (*p* = 0.008). Additionally, survival analysis demonstrated that up-regulated CRNDE expression is associated with poor overall survival of glioma patients (*p* < 0.001) [10]. Cox regression analysis revealed that HOXA-AS3 is an independent prognostic factor, and its high-expression is associated with tumor grade and poor prognosis in glioma patients [11].

As previously described, exosome-transmitted lncRNAs reflect the progress of the tumor microenvironment, which are stable and easily detectable in plasma or other body fluids and enable accurate diagnosis as desirable markers [66]. A potential application for minimally invasive liquid biopsy lies in the use of circulating lncRNA levels, which has been well documented in other cancers [67]. By comparing the expression of ten candidate circulating serum lncRNAs in a case-control study, three lncRNAs, LINC00152, RP11-160H22.5 and XLOC014172, have been identified as useful in the early diagnosis of hepatocellular carcinoma [68]. Moreover, lncRNAs with low expression levels in cells may be enriched in secreted exosomes for the regulation of cancer metastasis, thereby showing higher clinical utility [69].

## Therapeutics targeting tumor-specific lncRNA abnormalities

Since the modulation of lncRNA networks mediates anti-proliferative effects in malignant tumors and triggers therapeutic efficacy, targeting tumor-specific lncRNA abnormalities using small interfering RNAs (siRNAs) may be an effective strategy [70].

Many epigenetic inhibitors are currently used in clinical trials. For instance, bromodomain and extraterminal (BET) domain proteins reduce the levels of several oncogenes in glioblastoma, including lncRNA HOTAIR, and the use of BET inhibitor can restore the malignancy caused by oncogenic lncRNAs [71].

Antisense oligonucleotides (ASOs) can correct aberrant lncRNA networks by inducing RNase H-dependent degradation or sterically blocking lncRNA activity. For example, intravenous treatment with ASOs targeting lncRNA TUG1 coupled with a drug delivery system induces glioma stem cell differentiation and efficiently represses tumor growth in vivo [47]. Furthermore, chemical modifications of ASOs, such as the addition of 2′-omethyl and locked nucleic acids at both 5′ and 3′ ends, can protect ASOs from degradation and maintain sufficient concentrations. A preclinical study demonstrated that the use of modified ASOs targeting MALAT1 in a breast cancer mouse model promotes cystic differentiation and decreases tumor growth [72].

To make “anti-ncRNA therapy” more thermally stable in the tumor microenvironment, specific nucleic acids conjugated with a neutral-charged peptide backbone, called peptide nucleic acids (PNAs), are modified for in vivo applications [73]. PNAs-based strategy blocks the ability of lncRNA HOTAIR to interact with EZH2, subsequently reduces HOTAIR-EZH2 activity and inhibits ovarian and breast cancer cell invasion, which may be suitable for other solid cancers, such as glioma [74].

With a better understanding of the lncRNA binding domain, the use of small molecule inhibitors to disrupt the interactions of lncRNAs with their binding partners, particularly the chromatin modification complex, is an alternative strategy, which has been demonstrated for miRNAs [75]. However, given the length of some lncRNAs, it is possible that extensive secondary structures will restrict the accessibility of small molecule inhibitors to crucial parts of target lncRNAs, again emphasizing the importance of structural mapping for lncRNAs [19].

## Conclusion

At present, tens of thousands of identified lncRNAs have been associated with imbalanced gene regulation and aberrant biological processes in cancers [3, 6], and many lncRNAs play indispensable roles in the onset and progression of glioma malignancy, including the stemness, proliferation, angiogenesis and drug resistance [7, 8].

However, only a few functional mechanisms of lncRNAs have been well characterized, and some recent insights into lncRNAs identified in other diseases and cancers, such as molecules encoding tumor-related micropeptides, changes in the activities or conformation of proteins and the regulation of tumor microenvironment homeostasis, have not yet been clearly demonstrated in glioma pathogenesis. The majority of studies of glioma-associated lncRNAs are typically focused on ceRNA regulatory networks; thus, there is an urgent need to elucidate the detailed mechanisms of the roles of lncRNAs in glioma. Additionally, whether lncRNA expression in glioma is dysregulated and abnormal remains elusive, and the underlying mechanisms are still unknown.

Since lncRNAs mainly exert their functions by interacting with other biomolecules, the secondary structures of the interaction domains are of particular importance, and we anticipate that further investigations will focus on resolving lncRNA-binding motifs, which could yield new RNA-based targets for the prevention and treatment of this disease.

The lncRNA-based therapeutic strategies currently remain limited in vivo application, due to the relatively poor stability and intracellular uptake of drugs. Exosome delivery systems can greatly increase their bioavailability by preserving integrity, and the engineering of ligand-dependent exosome membranes that target specific brain tissues is also drawing interest, making therapy more efficient and target-specific.

## Abbreviations

Akt: Protein kinase B
ALKBH5: A-ketoglutarate-dependent dioxygenase alkB homolog 5
ASO: Antisense oligonucleotide
Bcl-x: B-cell lymphoma x
BET: Bromodomain and extraterminal
BRK: Breast tumor kinase
BTB: Blood–tumor barrier
CASC2: Cancer susceptibility candidate 2
circRNA: Circular RNA
ciRNA: Circular intronic RNA
CRNDE: Colorectal neoplasia differentially expressed
CSC: Cancer stem-cell-like
CXCR7: Chemokine receptor 7b
EMT: Epithelial-to-mesenchymal transition
EOC: Epithelial ovarian cancer cell
eRNA: Enhancer-associated RNA
ERV: Endogenous retrovirus
ESM-1: Endothelial cell-specific molecule 1
EZH2: Enhancer of zeste homolog 2
FLC: *FLOWERING LOCUS C*
FOXM1-AS: Forkhead box protein M1 antisense transcript
GBM: Glioblastoma multiform
GECs: Glioma-associated endothelial cells
GSCs: GBM stem-like cells
H3K27me3: Histone 3 lysine 27 trimethylation
H3K56ac: Histone 3 lysine 56 acetylation
HIF1α: Hypoxia-inducible factorα
hnRNP: Heterogeneous nuclear ribonucleoprotein
HOTAIR: HOX transcript antisense RNA
HOXA11-AS: Homeobox protein Hox-A11 antisense RNA 1
HULC: Human universal load carrier
lincRNAs: Intergenic lncRNAs
LINK-A: Long intergenic noncoding RNA for kinase activation
LncRNAs: Long non-coding RNAs
MALAT1: Metastasisassociated lung adenocarcinoma transcript 1
MEG3: Maternally expressed gene 3
MMP 3: Matrix metalloproteinase 3
MTA2: Metastasis-associated protein 2
mTOR: Mechanistic target of rapamycin
NEAT1_2: Nuclear enriched abundant transcript 1 the long isoform
ORF: Open reading frame
PI3K: Phosphatidylinositol-4,5-bisphosphate 3-kinase
PRC2: Polycomb repressive complex 2
PROMPT: Promoter upstream transcript
PTBP1: Polypyrimidine tract-binding protein 1
RISC: RNA-induced silencing complex
RNase P: Ribonuclease P
SERCA: Sarcoplasmic reticulum Ca^2+^-ATPase
SHP: Small heterodimer partner m6A N6-Methyladenosine
siRNA: Small interfering RNA
SIRT6: Sirtuin-6,
Snail: Snail1
SNHG5: Small nucleolar RNA host gene 5
sno-lncRNA: SnoRNA-ended lncRNA
snoRNA: Small nucleolar RNA
SPA: 5’ SnoRNA-ended and 3′-polyadenylated lncRNA
SPAR: Small regulatory polypeptide of amino acid response
TAM: Tumor associated macrophage
TERRA: Telomeric repeat-containing RNA
TMZ: Temozolomide
TrxG: Trithorax Group proteins
TUG1: Taurine upregulated gene 1
TUSC7: Tumor suppressor candidate 7
USF: Stimulating factor
XIST: X-inactive specific transcript
